# Full-Scale Aggregated MobileUNet: An Improved U-Net Architecture for SAR Oil Spill Detection

**DOI:** 10.3390/s24123724

**Published:** 2024-06-07

**Authors:** Yi-Ting Chen, Lena Chang, Jung-Hua Wang

**Affiliations:** 1 Department of Electrical Engineering, National Taiwan Ocean University, Keelung 202301, Taiwan; 20953003@mail.ntou.edu.tw (Y.-T.C.); jhwang@mail.ntou.edu.tw (J.-H.W.); 2 Department of Communications, Navigation and Control Engineering, National Taiwan Ocean University, Keelung 202301, Taiwan; 3 The Intelligent Maritime Research Center (IMRC), National Taiwan Ocean University, Keelung 202301, Taiwan; 4 Department of Electrical Engineering, AI Research Center, National Taiwan Ocean University, Keelung 202301, Taiwan

**Keywords:** oil spills, synthetic aperture radar (SAR), U-Net, semantic segmentation models

## Abstract

Oil spills are a major threat to marine and coastal environments. Their unique radar backscatter intensity can be captured by synthetic aperture radar (SAR), resulting in dark regions in the images. However, many marine phenomena can lead to erroneous detections of oil spills. In addition, SAR images of the ocean include multiple targets, such as sea surface, land, ships, and oil spills and their look-alikes. The training of a multi-category classifier will encounter significant challenges due to the inherent class imbalance. Addressing this issue requires extracting target features more effectively. In this study, a lightweight U-Net-based model, Full-Scale Aggregated MobileUNet (FA-MobileUNet), was proposed to improve the detection performance for oil spills using SAR images. First, a lightweight MobileNetv3 model was used as the backbone of the U-Net encoder for feature extraction. Next, atrous spatial pyramid pooling (ASPP) and a convolutional block attention module (CBAM) were used to improve the capacity of the network to extract multi-scale features and to increase the speed of module calculation. Finally, full-scale features from the encoder were aggregated to enhance the network’s competence in extracting features. The proposed modified network enhanced the extraction and integration of features at different scales to improve the accuracy of detecting diverse marine targets. The experimental results showed that the mean intersection over union (mIoU) of the proposed model reached more than 80% for the detection of five types of marine targets including sea surface, land, ships, and oil spills and their look-alikes. In addition, the IoU of the proposed model reached 75.85 and 72.67% for oil spill and look-alike detection, which was 18.94% and 25.55% higher than that of the original U-Net model, respectively. Compared with other segmentation models, the proposed network can more accurately classify the black regions in SAR images into oil spills and their look-alikes. Furthermore, the detection performance and computational efficiency of the proposed model were also validated against other semantic segmentation models.

## 1. Introduction

Oil spills pose a significant threat to the environment, affecting bodies of water, land, and the air [1]. Oil spill incidents, often caused by accidents involving oil tankers, ships, and pipelines, release crude oil, gasoline, fuel, and oil by-products into water bodies, thereby polluting water and harming aquatic life. Oil spills have increased due to intensive oil exploration and transportation, which are driven by global demand. The environmental and socio-economic impacts are severe, causing water pollution, shoreline degradation, and economic losses in fishing and marine industries [2].

In the past, conventional on-site monitoring played a significant role in oil spill detection. However, this monitoring carried various risks to those conducting it, including direct contact with oil and exposure to other site hazards [3]. Subsequently, ocean surveillance systems consisting of aircraft and coastguard forces were introduced. While these systems proved effective, the high cost associated with mapping extensive areas hindered their widespread adoption [4]. Synthetic Aperture Radar (SAR) mounted on aircraft or satellites plays a crucial role in detecting oceanic oil spills by emitting electromagnetic pulses and capturing reflected echoes. Therefore, SAR images are now the favored data source for oil spill detection owing to their high resolution, all-day observation, robust penetration ability, and extensive spatial coverage capabilities. In SAR images, oil on the sea surface can be considered a dark area because of the suppression of capillary waves and a reduction in radar backscatter. This results in the depiction of oil spills as black spots, contrasting with the brighter regions of uncontaminated sea areas [5,6]. However, challenges persist, including misclassification of dark spots and the presence of look-alikes such as low wind areas and algae blooms [7,8,9]. Despite the widespread use of SAR, there is a need for enhanced detection accuracy and minimized response time to address the global issue of oil spills. This study highlights the importance of early detection, monitoring, and timely intervention using SAR technology to mitigate the environmental disasters caused by oil spills and protect marine ecosystems.

In recent years, many studies have attempted to detect oil spills using SAR data. Typically, detection methods are classified into two categories, where the input SAR image is annotated as oil spills or look-alikes. Solberg et al. [5] proposed an automated detection algorithm with a three-phase process to identify oil spills and look-alikes, including prior knowledge, Gaussian density, and rule-based density corrections. Chang et al. [10] proposed a region-based SAR oil spill detection method. First, the segmentation method was used to extract the oil spill in SAR images, and an oil spill model was established. Finally, the generalized likelihood ratio test method was used to derive a closed-form solution for oil spill detection using this model. Karathanassi et al. [11] proposed an object-oriented approach and employed adaptive local contrast and brightness thresholds for image segmentation to identify dark formations. Two empirical formulas for oil spill classification based on brightness were established, considering the characteristics of dark areas and marine environments. Fuzzy classification methods were then applied to differentiate look-alikes. Konik et al. [12] proposed an efficient decision tree forest to evaluate important features that distinguish oil spills from look-alikes. Keramitsoglou et al. [13] introduced an automated system using artificial intelligence fuzzy logic to detect potential oil spills using SAR images. The system analyzes SAR images to identify dark patterns with characteristic shapes that indicate an oil spill. The output provides users with relevant information for decision-making through images and tables. Karantzalos et al. [14] proposed an approach involving a pre-processing step with advanced image simplification, followed by geometric level set segmentation to detect potential oil slicks. Finally, a classification is performed to separate look-alikes, resulting in the extraction of oil spills. Fiscella et al. [15] presented a probabilistic approach to distinguish oil spills from look-alikes based on the statistics of previously measured characteristics. Espedal et al. [16] improved the oil spill recognition system by incorporating historical wind data and estimating the duration of time since the spill occurred.

SAR sensors are capable of monitoring large areas and can, therefore, include additional contextual information, such as ships, coastal structures, platforms, and land. This contextual information is semantically significant in the classification process. For example, a dark spot with a linear formation near a ship indicates an oil spill from the ship rather than a look-alike. Moreover, detailed information about nearby coastal areas or ships is crucial for early warning systems and decision-making modules to mitigate the overall risk. Consequently, a segmentation approach is necessary to accurately identify multi-class instances in SAR images. Furthermore, oil spills across the sea surface are dynamic and evolving phenomena influenced by factors like wind speed and sea currents, resulting in oil slicks of various shapes and sizes to account for these variations and eliminate the need for handcrafted features. These techniques can evaluate geometric characteristics such as shape and size. Considering these factors, along with the presence of multi-class instances, semantic segmentation models become a robust alternative capable of extracting rich informative content from SAR images. Therefore, the development of an automated oil detection model capable of classifying these elements can enhance the overall detection performance. Implementing a neural network for the early detection of oil spills, whether in specific regions or on a broader scale, could provide timely alerts to relevant authorities, expediting responses to such disasters. Therefore, this study utilized deep learning to extract features from SAR images. These models contribute to decision-making processes through semantic segmentation, particularly in classifying oil spills.

In the past few years, the utilization of convolutional neural networks (CNNs) has surpassed the performance of exclusively relying on traditional methods in various tasks and applications, even for the remote sensing data [17]. Deep convolutional neural networks, particularly in the case of fully convolutional networks (FCNs), demonstrate superior performance in extracting semantic image features for detection purposes [18]. In response to the difficulty of overlooking global context information in the FCN method, Zhao et al. [19] introduced the Pyramid Scene Parsing Network (PSPN), a multi-scale network crafted to enhance the effective capture of a scene’s global contextual representation. Moreover, popular image segmentation models based on the encoder–decoder structures, such as U-Net [20] and DeepLab series [21,22], have been utilized for oil spill segmentation. Basit et al. [23] used EfficientNetb0 as the encoder backbone of U-Net for multi-class classification, including oil spills, look-alikes, land, sea surface, and ships. Fan et al. [24] proposed a feature merge network (FMNet) based on the combination of threshold segmentation algorithms and U-Net to extract the global features of oil spills using SAR images. Rousso et al. [25] used a SAR image filtering technique to emphasize the physical characteristics of oil spills. Subsequently, the detection performance was improved in U-Net and DeepLabv3+ architectures. Shaban et al. [26] introduced a two-stage deep learning framework designed for oil spill detection, particularly focusing on a highly unbalanced dataset. In the first stage, a novel 23-layer CNN classifies patches by considering the percentage of pixels associated with an oil spill. The second stage employs a five-stage U-Net structure for semantic segmentation. Mahmoud et al. [27] introduced a novel deep learning model for the automated detection of oil spills using the Dual Attention Model (DAM). The U-Net segmentation network was improved by integrating DAM, allowing the selective highlighting of local and global characteristics in SAR images. DAM consists of two components, namely the Channel Attention Map and the Position Attention Map, which were integrated into the decoder part of the U-Net. Li et al. [28] proposed a dual-stream U-Net (DS-UNet) for SAR oil spill detection. The proposed model consisted of two parts: one focused on inter-scale alignment for the extraction of global information, and one focused on feature extraction of edges to capture local information. Moreover, Ma et al. [29] used the Sentinel-1 dual-polarimetric data for oil spill detection and incorporated amplitude and phase information. In addition, the Cloude polarimetric decomposition parameters were integrated into the proposed model to enhance feature extraction. The experimental results showed that the proposed modified DeepLabv3+ model with ResNet101 as the backbone can improve detection performance.

The U-Net model with a simple and effective encoder–decoder architecture and skip connections method make it a powerful tool in semantic segmentation models, especially when working with small datasets and requiring high-resolution outputs. The above studies [23,24,25,26,27,28,29] showed that the U-Net model could achieve better detection performance in oil spill detection within semantic segmentation models. However, the detection results showed that some oil spill regions were incomplete, fragmented, and misclassified as look-alikes, resulting in the degradation of detection performance. The correct and effective classification of black areas is crucial to improving the oil spill detection performance. Therefore, the study adopted the U-Net architecture and further improved the encoding and decoding layers to effectively extract features of oil spills and achieve better discrimination from look-alikes. In this study, an improved lightweight U-Net model, Full-Scale Aggregated MobileUNet (FA-MobileUNet), was introduced to improve the oil spill detection performance using SAR images. Due to the distinct characteristics of various marine targets, this study focused on modifying the network architecture to improve feature extraction. The modified network enhanced the extraction of features and aggregated features at different scales to improve the accuracy of detecting diverse marine targets. First, the U-Net encoder was replaced with MobileNetv3 as the backbone network to improve feature extraction while reducing the number of parameters. Next, atrous spatial pyramid pooling (ASPP) and a convolutional block attention module (CBAM) were utilized to improve the feature extraction while reducing the computational burden. These improvements enhanced the detection speed and prevented the loss of target information to obtain more comprehensive semantic features. Finally, the capacity of the network was improved by aggregating low-level and high-level features at different scales to enhance segmentation accuracy.

The rest of the paper is structured as follows: Section 2 describes the data used in this study. Then, the method of the proposed model is introduced. The experimental results and discussions are presented in Section 3 and Section 4, respectively. Finally, conclusions are drawn in Section 5.

## 2. Materials and Methods

### 2.1. Oil Spill Dataset

#### 2.1.1. MKLab Dataset

Due to the difficulty in obtaining SAR images of oil spills, the detection of oil spills remains a challenging issue. Moreover, the lack of a common dataset for oil spill detection is a major limitation that must be addressed. Previous studies [12,27,28,29,30] used different custom datasets that corresponded to the specific methodologies used at the time. Nevertheless, the results presented lack comparability because each deep learning-based semantic segmentation approach employed a distinct dataset, preventing the establishment of a common basis for comparison.

In 2019, Krestenitis et al. [31] created a common dataset for oil spill detection using SAR images, and it is publicly accessible on their website (https://mklab.iti.gr/results/oil-spill-detection-dataset/, accessed on 24 January 2022). In brief, SAR images featuring areas of the sea contaminated with oil were collected from the European Space Agency (ESA) database, specifically the Copernicus Open Access Hub (https://scihub.copernicus.eu/, accessed on 24 January 2022). Geographical coordinates and timestamps for pollution events were provided by the European Maritime Safety Agency (EMSA) through the CleanSeaNet service. Consequently, the identification of dark areas in the SAR images as oil spills was corroborated by the EMSA records, establishing robust ground truth data. The oil spills recorded span from September 2015 to October 2017, while the SAR images were sourced from the Sentinel-1 European Satellite missions. Sentinel-1 satellites utilize a SAR system operating at C-band, offering a ground range coverage of approximately 250 km and a pixel spacing of 10 × 10 m. The radar image polarization is dual, with VV and VH polarizations. For the SAR oil spill dataset, only the raw data from the VV polarization were processed.

**Figure 1:**
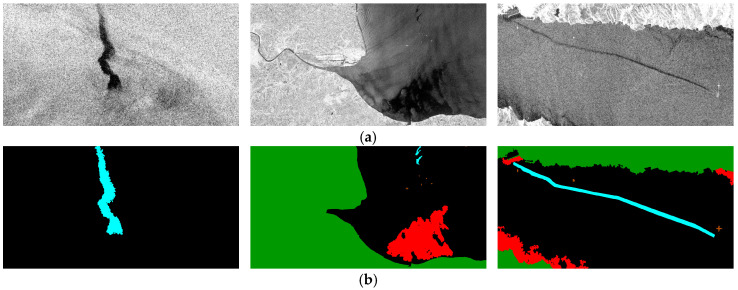
Samples of SAR oil spill images from the MKLab dataset. Cyan, red, brown, green, and black correspond to oil spills, look-alikes, ships, land, and sea surface, respectively. (**a**) SAR images. (**b**) RGB masks.

After preprocessing by the authors [31], the dataset contained 1112 images with a resolution of 1250 × 650, which were divided into 1002 training and 110 testing images. The dataset contained a total of five categories, including oil spills, look-alikes, ships, land, and sea surface, and each category was assigned a different RGB color, as shown in Figure 1. The RGB labels were created for the images, with cyan, red, brown, green, and black masks corresponding to oil spills, look-alikes, ships, land, and sea surface, respectively. RGB masks were mainly used for different categories to support visualization. However, for the deep learning training and evaluation processes, one-dimensional target labels were required instead of RGB values. Therefore, one-channel label masks were also provided by assigning each color category an integer value from 0 to 4, as shown in Table 1.

#### 2.1.2. MKLab Dataset Augmentation

Due to the inherent challenges in oil spill detection based on SAR imagery, the data were distributed differently among the categories. Typically, samples from the sea or land category dominate the dataset, while data on oil spills, look-alikes, and ships are often confined to smaller regions in the SAR images. Moreover, insufficient data can lead to overfitting and poor generalization of the deep learning network. Therefore, this study augmented the MKLab dataset by searching for marine oil spill events and obtaining Sentinel-1 images from the ESA website based on the location and time of the events. Spanning from 2015 to 2022, 127 SAR images containing marine oil spills were collected to augment the dataset for the subsequent deep learning model training process. The study annotated the acquired SAR images according to the format in Table 1 to ensure consistency with the labeled data in the MKLab dataset.

**Figure 2:**
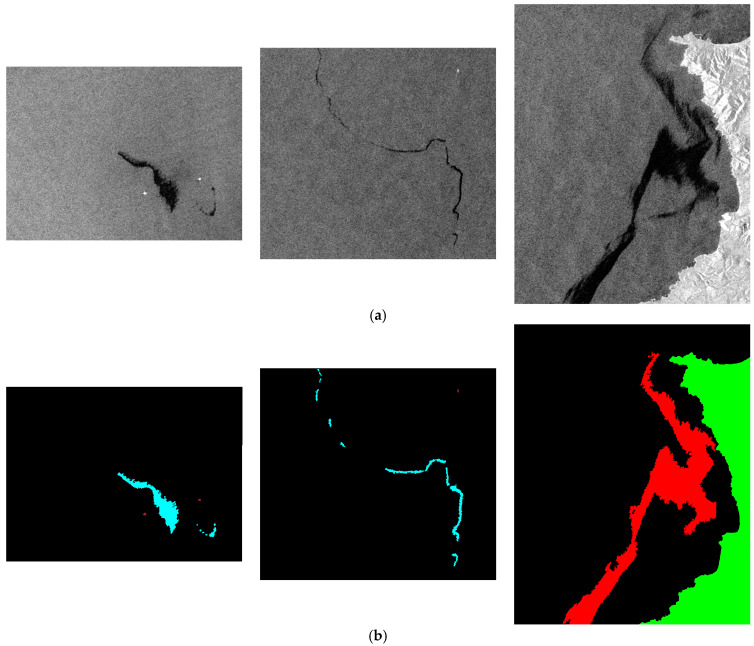
The collected SAR images corresponding to oil spill events in the Mediterranean Sea. The sampling dates from left to right are 25 February 2021, 5 September 2021, and 5 September 2021. Cyan, red, brown, green, and black correspond to oil spills, look-alikes, ships, land, and sea surface, respectively. (**a**) SAR images. (**b**) RGB masks.

Therefore, this study used a hierarchical image segmentation algorithm supplemented with manual inspection to label different categories, as shown in Figure 2. The first stage separated the sea surface from the land. The image was binarized through grayscale morphological opening and closing operations, as well as through Otsu’s thresholding method. Since the average gray scale of the land backscattering was higher than that of the sea, the land part was expected to correspond to a white area. However, there were some small black regions inside; conversely, the sea part was a large black area containing small white regions. Then, through the morphological hole filling, the small black holes existing in the large white region were filled, so that the land part corresponded to the white region. The sea part was processed through negative conversion first, and the sea part was inverted into a large white region, which contained some small black regions. Then, the small black regions in the sea were filled with closing technology. After negative conversion, the sea part corresponded to the black region. This process effectively separated the sea from the land, with the land labeled in green. The second stage involved segmenting the ship targets in the sea area. Targets with strong reflections in the sea are suspected ship targets. Therefore, the non-ship targets were first removed through edge and line detection. After smoothing through morphological binary image dilation and erosion, edge detection was performed using the Sobel edge detection algorithm [32]. Subsequently, non-ship targets arranged in a straight line were removed through straight line detection. Ship target identification was then conducted by comparing their characteristics (size, shape, and statistical), and the ship targets were labeled in brown. The last step served to segment the oil spills and look-alikes in the sea. Oil spills on the sea surface suppress waves, resulting in weaker radar backscatter and darker gray scale in images. In the leeward part of the sea or when the wind speed is low, darker gray scales similar to oil spills appear on SAR images. Therefore, the research used this feature to cut out the dark gray-scale area from the sea, and then compare the gray-scale contrast between this area and the surrounding sea area. According to the geo-location of the oil pollution incident, supplemented by manual interpretation, oil spills and look-alikes were distinguished and labeled in cyan and red, respectively. Finally, the different colored categories were converted into annotations ranging from 0 to 4. Additionally, the annotation method was employed to verify the ground truth data of the MKLab dataset, ensuring that the ground truth data of the images were correct.

### 2.2. The Proposed FA-MobileUNet Model

To achieve high-performance oil spill detection, deep learning networks are used to extract the unique features of oil spills in SAR images. However, marine oil spill SAR images may cover multiple categories, including oil spills, look-alikes, sea surface, land, and ships. The distribution of the data in the MKLab dataset was highly unbalanced. Therefore, this study introduced a semantic segmentation model to improve detection performance with a limited training dataset. This study proposed a lightweight segmentation network structure called the Full-Scale Aggregated MobileUNet (FA-MobileUNet) model to address oil spill detection, with the purpose of improving segmentation precision and reducing mis-segmentation between oil spills and look-alikes. Effectively extracting the adjacent spatial information of the black areas in images enables deep learning models to be more accurate when learning the features of oil spills and look-alikes. In addition, various ocean phenomena result in diverse scales for black areas. Therefore, it is important to improve the model spatial and multi-scale feature extraction capabilities.

**Figure 3:**
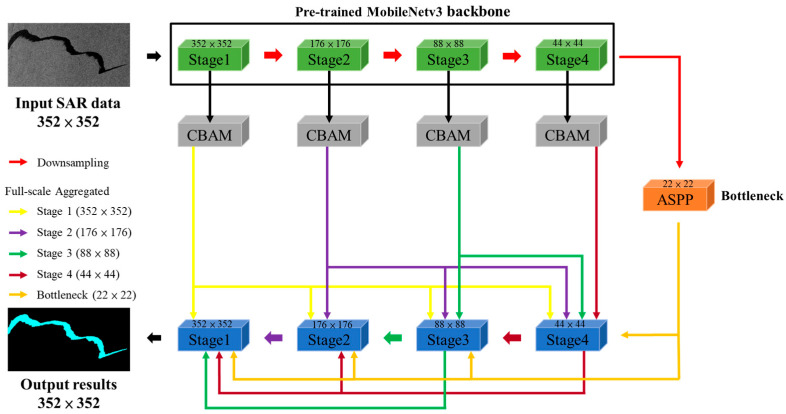
The architecture of the proposed FA-MobileUNet model.

The proposed network was designed as an end-to-end structure comprising an encoder–decoder architecture. First, in order to reduce the computation cost of the model and effectively extract feature maps, MobileNetv3 was used as the backbone architecture of the model. Next, ASPP and CBAM were added to allow the model to extract complex image details and provide multi-scale contextual information. Finally, the full-scale aggregation architecture facilitated the connection of low-level spatial information and high-level semantic features at various scales, enhancing the extraction of contextual information. These modules could reduce the misclassification of dark areas and prevent the fragmentation of detection results. Therefore, the proposed model effectively distinguished oil spills in SAR images and improved the accuracy of oil spill detection through the improvement of feature extraction. The overall architecture of the proposed FA-MobileUNet model is shown in Figure 3.

#### 2.2.1. U-Net

**Figure 4:**
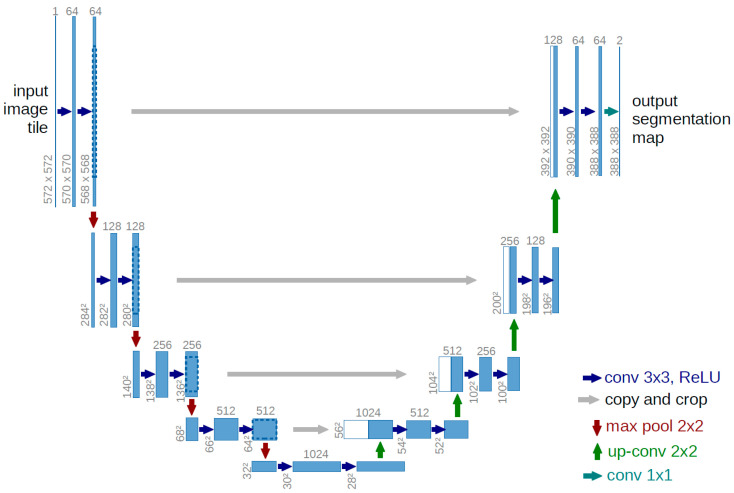
The U-Net structure proposed by Ronneberger et al. [20].

U-Net [20], originally introduced by Ronneberger et al. in 2015, is an extension of the FCN structure developed for biomedical image semantic segmentation. It has gained widespread adoption across various applications. The architecture of the U-Net model consists of a five-stage contraction stage (encoder), a five-stage expansive stage (decoder), and a bottleneck bridge, as shown in Figure 4. The encoder part adopts an FCN-based architecture to capture the image’s content. In contrast, the decoder part facilitates precise localization by upsampling the extracted feature map while reducing its filters, creating a broader but shallower representation. Each block of the encoder consists of two 3 × 3 convolutional layers with a Rectified Linear Unit (ReLU) activation function, followed by a maxpooling layer with a 2 × 2 kernel size and stride of 2. The number of channels in the five stages of the encoder is as follows: 64, 128, 256, 512, and 1024. The decoder consists of an upsampling layer followed by a 3 × 3 convolutional layer, a concatenate layer with features from the corresponding path of the encoder, two 3 × 3 convolutional layers and ReLU activation, and a maxpooling layer with a kernel size of 2 × 2 and stride of 2. Finally, the output from the decoder is subsequently processed through a 1 × 1 convolution employing the Sigmoid activation function to derive the probability of class prediction for each pixel.

#### 2.2.2. MobileNetv3

MobileNet introduces numerous innovative concepts aimed at minimizing the number of parameters, making it more efficient for mobile devices while simultaneously achieving high classification accuracy. MobileNetv1 [33] was first introduced in 2017 and designed to optimize accuracy while considering the limited resources of on-device or embedded applications. MobileNetv1 successfully achieves two primary objectives: reducing model size and complexity to create efficient computer vision models for mobile applications. The basic architecture of MobileNetv1 relies on an efficient design that uses depth-wise separable convolutions to build lightweight deep neural networks. Next, the second version of the MobileNet architecture was introduced in 2018 [34]. MobileNetv2 incorporates new elements to optimize the architecture for tasks such as classification, object detection, and semantic segmentation. MobileNetv2 introduces two new features to the architecture: shortcut connections between the bottlenecks and linear bottlenecks between the layers. The fundamental concept behind MobileNetv2 is that the bottlenecks encode the intermediate inputs and outputs of the model, while the inner layer encapsulates the model’s ability to transform from lower-level concepts, such as pixels, to higher-level descriptors like image categories. Similar to traditional residual connections, these shortcuts help make training faster and improve the accuracy. The latest advancements in the MobileNet architecture were consolidated and documented in 2019 [35]. The key innovation of MobileNetv3 is the use of AutoML (Automated Machine Learning) to identify the optimal neural network architecture for a given problem. MobileNetv3 initially employs MnasNet, a reinforcement learning-based approach, to explore a coarse architecture by selecting the most suitable configuration from a set of discrete choices. Thereafter, the model refines the architecture using NetAdapt, an additional technique that gradually trims under-utilized activation channels. A distinctive feature of MobileNetv3 is the integration of squeeze-and-excitation (SE) blocks [36] into the core architecture. These blocks enhance the quality of representations produced by the network by explicitly modeling interdependencies between channels in its convolutional features. In the context of MobileNetv3, this architecture extends MobileNetv2 by incorporating SE blocks into the search space, resulting in more robust architectures. Furthermore, the implementation of the hard-swish activation function enhances expressiveness while preserving computational efficiency, achieving a crucial balance in capturing complex data without sacrificing performance.

**Figure 5:**
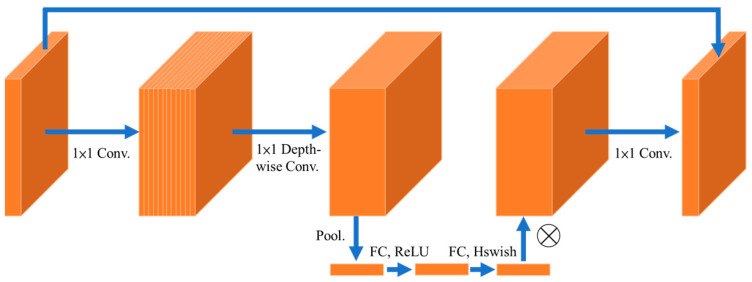
The block structure of MobileNetv3.

The limited number of images in the oil spill dataset poses a challenge in training deep models from scratch because it can easily lead to overfitting issues. To overcome this challenge, the proposed model mitigates overfitting by incorporating a pre-trained backbone network. Tuning a pre-trained model through transfer learning is a common practice in machine learning and can produce superior results than training it from scratch, particularly when dealing with small datasets. Therefore, MobileNetv3 was used as the backbone architecture of the proposed model in this study, as shown in Figure 5. The backbone network was pre-trained on the ImageNet dataset, which had more than 14 million samples. This advantage made the backbone of the proposed model highly effective in extracting descriptive feature maps.

#### 2.2.3. Attention Mechanism

The attention mechanism is a key component of many deep learning methods, and was designed to improve the model’s ability to focus on relevant parts of the input data. In the context of neural networks, attention mechanisms allow models to assign different levels of importance to different parts of the input image. Rather than processing the entire input equally, the model can selectively attend to specific regions or features that are more relevant to the task at hand, enabling it to capture long-range dependencies and improve its performance in complex tasks.

**Figure 6:**
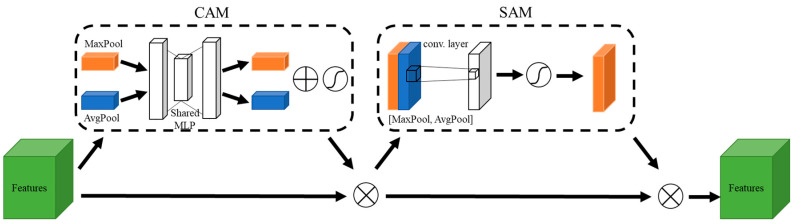
The structure of the CBAM.

The CBAM [37] is particularly useful for enhancing feature representation and improving the model’s ability to focus on informative spatial and channel-wise features. The CBAM typically consists of two attention sub-modules: the channel attention module (CAM) and the spatial attention module (SAM), which are shown in Figure 6. The CAM module focuses on capturing inter-channel dependencies by computing a channel-wise attention map, allowing the model to emphasize important channels and suppress less relevant ones. The SAM module captures intra-channel dependencies and computes a spatial attention map, which helps the model focus on specific spatial locations within each channel, highlighting important regions. Therefore, the combination of channel and spatial attention allows the CBAM to adaptively recalibrate the feature maps at different levels of abstraction, enabling the model to capture more informative and discriminative features. This capability is particularly valuable when dealing with SAR oil images where categories vary in size, shape, and context. Therefore, the output feature maps from the encoder of the proposed model were enhanced through the CBAM.

#### 2.2.4. Atrous Spatial Pyramid Pooling (ASPP)

**Figure 7:**
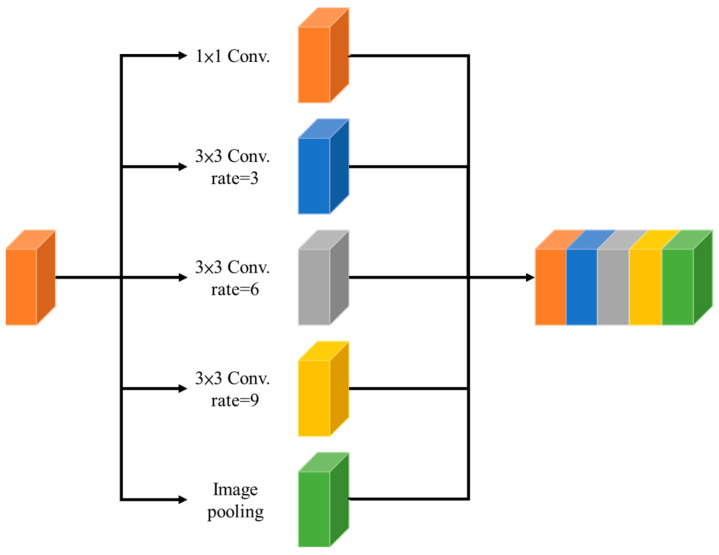
The structure of the ASPP module.

The ASPP module was first proposed in the semantic segmentation network DeepLabv2 [21]. ASPP serves as a pivotal feature extraction module in the domain of CNNs, playing a crucial role in semantic segmentation tasks. The goal is to integrate global contextual information across multiple scales within an image, all while avoiding downsampling of input feature maps. The core of ASPP is the concept of atrous convolutions, also known as dilated convolutions. This unique property introduces gaps between filter weights, enabling the model to capture features from broader spatial information without compromising on the resolution of the input feature map. This design decision allows the network to capture context information at different scales, with lower dilation rates catering to finer details and higher rates addressing more global contextual features. An integral component of ASPP is the inclusion of an image-level feature obtained through global average pooling. This feature summarizes the entire feature map, providing the model with a holistic understanding of the image content. This diversity of features processed at different scales and with varying levels of context information enriches the overall feature representation, making ASPP particularly effective in multi-category semantic segmentation tasks. Therefore, in the proposed model, the bottleneck bridge between the encoder and decoder utilized the ASPP module to improve the detection performance. The structure of the ASPP module is shown in Figure 7. The dilation rates of ASPP were selected as 1, 3, 6, and 9 in this study, respectively.

#### 2.2.5. Full-Scale Aggregation

**Figure 8:**
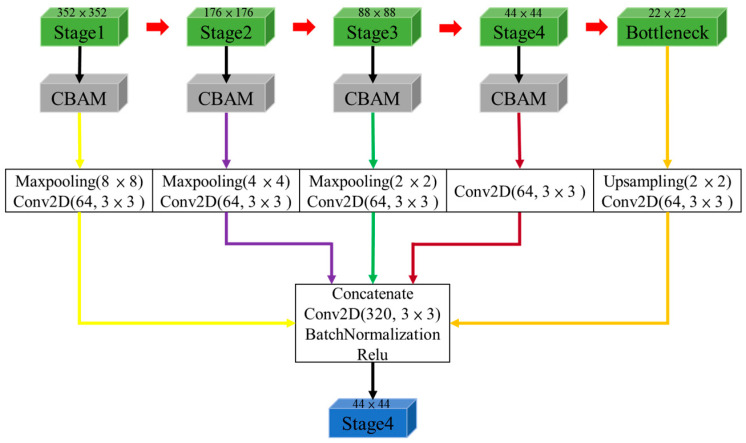
Full-scale aggregation example of stage 4 (44 × 44) of the decoder layer in Figure 3.

Low-level detail feature maps capture spatial information, emphasizing the target boundaries, whereas high-level semantic feature maps express positional information, indicating the locations of targets. However, these meaningful features may gradually disappear when progressively upsampling and downsampling. The Full-Scale Aggregation (FA) network module represents a significant advancement in the field of computer vision, particularly in semantic segmentation. Its purpose is to effectively extract multi-scale contextual semantic information within CNNs. Therefore, the FA module can capture contextual features at various scales from the U-Net encoder and aggregate the output features of the encoder from different levels with the U-Net decoder, as shown in Figure 8. In the present study, the encoder output feature maps enhanced through the CBAM were sampled to the same scale as the decoder by the upsampling/maxpooling layer followed by a 3 × 3 convolutional layer with 64 filters. Subsequently, feature fusion was performed through the concatenate operation. Therefore, the FA combined feature maps from different levels of the encoder, which can capture coarse-grained semantics and fine-grained details at full scale.

### 2.3. Evaluation Metric

In the evaluation process, the Intersection over Union (IoU) and F1-score was utilized to evaluate the performance of the semantic segmentation network. IoU is defined as the ratio of the area of overlap between the predicted region and the ground truth region to the area of union between these two regions. The F1-score is a comprehensive indicator that combines precision and recall to evaluate the performance of different semantic segmentation models. The formulas are as follows:

(1) $$IoU=\frac{Ground\;truth\cap Prediction}{Ground\;truth\cup Prediction}=\frac{TP}{TP+FP+FN}$$

(2) $$Precision=\frac{TP}{TP+FP}$$

(3) $$Recall=\frac{TP}{TP+FN}$$

(4) $$F1\text{-}score=2\times \frac{Precision\times Recall}{Precision+Recall}$$

where TP (true positive) represents the model correctly identifying and classifying positive examples; FP (false positive) represents the model incorrectly identifying an example as belonging to the positive class; FN (false negative) represents the model failing to identify an example that belongs to the positive class. In the experiments, IoU was measured for each category in the dataset, and the mean IoU (mIoU) was computed as the average values of IoU across all categories.

## 3. Results

### 3.1. Experimental Settings

The study conducted a series of experiments on the MKLab dataset and compared the detection performance with other segmentation models to verify the efficiency of the proposed model in this section. The experiments were performed on a PC equipped with a 12th Gen Intel Core i7-12700KF CPU with 16 GB of memory, 12 GB memory of NVIDIA RTX3080, and using CUDA 12.1 with cuDNN v8.8.0. The operating system was Windows 10 with a 64-bit processor. In addition, Tensorflow-gpu (version 2.10.1) and Keras (version 2.10.1) were used. In this study, the proposed FA-MobileUNet model was modified based on the open-source U-Net [38] architecture.

**Figure 9:**
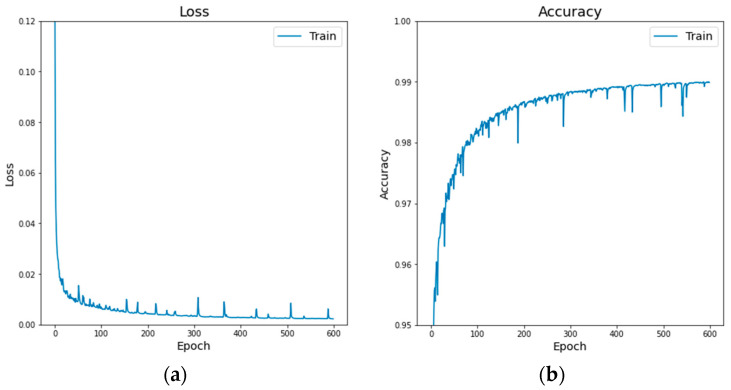
The training process of the proposed FA-MobileUNet model using augmented MKLab dataset. (**a**) Loss. (**b**) Accuracy.

The training set of the original MKLab dataset was augmented with the 127 collected SAR images to improve the generalization ability and efficiency of the deep learning model. Therefore, two datasets were used for the detection performance comparison, including the original MKLab dataset and the augmented MKLab dataset, which consisted of 1112 and 1239 images, respectively. In the training step, the number of epochs and the batch size were set as 600 and 8, respectively. The input image size was set as 352 × 352. The learning rate of the model was set as 5 × 10^−5^. In addition, the Adam [39] optimization method and categorical cross-entropy function were selected to train the models. As shown in Figure 9, the proposed FA-MobileUNet model using the augmented MKLab dataset achieved an accuracy of 0.9901, with a loss of 0.0016.

### 3.2. Ablation Experiments

Through the ablation experiments in this section, the modules of the proposed FA-MobileUNet model were evaluated on the augmented MKLab dataset and compared with the baseline U-Net model. The following experiments were conducted on all possible combinations of the CBAM, ASPP, and FA modules to observe their respective efficiencies in enhancing the detection performance, as shown in Table 2.

First, by separately integrating the CBAM, ASPP, and FA modules into the original U-Net model, the mIoU can be increased by 2.88%, 4.39%, and 6.95%, respectively, compared with the original U-Net model. When comparing the three modules individually, the ASPP and FA modules effectively captured feature maps at different scales, thereby improving the detection performance. In particular, for U-Net with the FA module, a significant improvement in mIoU was achieved with just a 1% increase in the number of parameters. Next, the experiments compared paired combinations of modules. Moreover, combining the CBAM and ASPP modules achieved a mIoU of 78.42%, which was 0.72% lower than that of only using the FA module. However, combining the FA module with the other two modules achieved a mIoU of over 80%. Finally, the original U-Net combined with all three modules reached the highest detection performance with an mIoU of 82.37%. Compared with the original U-Net model, the number of parameters increased by approximately 30% and the mIoU improved by approximately 10%. The experiment results validated the performance improvement when using the proposed modules.

### 3.3. Accuracy Assessment Based on Different Backbone Models

To verify the lightweight pre-trained backbone models, the encoder of the original U-Net was replaced with different CNN backbone architectures to evaluate the detection performance using the augmented MKLab dataset, including VGG16 [40], VGG19 [40], ResNet50 [41], DenseNet121 [42], EfficientNetB0 [43], Inceptionv3 [44], MobileNetv2 [34], and MobileNetv3 [35], as shown in Table 3. The original U-Net model had 31.03 M parameters and reached an mIoU of 72.19%. Replacing the backbone network with VGG16 and VGG19 architectures reduced the number of parameters by 5.17 M and 2.22 M, respectively. However, the detection performance did not improve much, with mIoUs of 72.73% and 72.82%, respectively. Using the ResNet50 backbone network, the number of parameters decreased to 20.67 M, and the mIoU dropped to 67.21%. In addition, replacing the backbone network with a DenseNet121, EfficientNetB0, Inceptionv3, MobileNetv2, or MobileNetv3 architecture reduced the number of parameters by more than 40%. Among them, the U-Net model with the DenseNet121 or Inceptionv3 backbone architecture had similar detection performance, with mIoUs of 74.77% and 75.22%, respectively. Moreover, the MobileNet backbone architecture can effectively reduce the number of parameters while maintaining high detection performance. The U-Net model with a MobileNetv3 backbone architecture achieved a better detection performance than the U-Net model with an Inceptionv3 backbone architecture, reaching an mIoU of 75.98%. Furthermore, the number of parameters was only 10.57 M. All backbone architectures adopted in these experiments were pre-trained on the ImageNet dataset. By using these efficient backbone networks with the pre-trained weights, the U-Net model not only trained faster but also had improved feature extraction. Compared with the original U-Net model, the model with a MobileNetv3 backbone had a reduction in the number of parameters of approximately 66% and the mIoU increased by 3.79%. The experimental results validated that the U-Net model with the pre-trained MobileNetv3 backbone architecture can effectively reduce the number of parameters and improve the detection performance.

### 3.4. Segmentation Network Comparison

In this section, the authors compare the detection performance of the proposed FA-MobileUNet model with other segmentation network architectures, including U-Net [31], LinkNet [31], PSPNet [31], DeepLabv2 [31], DeepLabv3+ [31], ToZero FMNet [24], the Ensemble model [25], CoAtNet-0 [45] and EfficientNetv2 [45]. Krestenitis et al. [31] used different semantic segmentation models and replaced the backbone network with ResNet-101 and MobileNetv2 for testing. Fan et al. [24] combined the feature merge network (FMNet) and the threshold segmentation algorithm based on the U-Net model to extract more semantic features. Rousso et al. [25] combined the U-Net and DeepLabv3+ models with different filtering algorithms and conducted ensemble training to enhance the models’ generalization capabilities. Basit et al. [45] introduced a new gradient profile (GP) loss function and combined it with other loss functions to improve the detection performance.

First, the detection performance of the proposed model was compared with that of the deep learning models in [31] using the original MKLab dataset. Table 4 summarizes the performance evaluation of the different semantic segmentation models in terms of IoU. The U-Net, LinkNet, PSPNet, and DeepLabv2 models all utilize the ResNet-101 backbone network. The detection results showed that the performance of U-Net and LinkNet improved, while the performance of PSPNet worsened slightly, and DeepLabv2 had the worst performance. In addition, compared with the U-Net using the ResNet-101 backbone network, DeepLabv3+ with a MobileNetv2 backbone network achieved a better detection performance, reaching an mIoU of 65.06%. However, the FA-MobileUNet achieved an mIoU of 78.93%, which was 13.96% and 13.87% higher than that of the U-Net and DeepLabv3+ models, respectively. Compared with the original U-Net model, the IoU of oil spills and look-alikes using the FA-MobileUNet model increased by 16.63% and 30.60%, respectively.

Next, the deep learning models presented in Table 4 were trained using the augmented MKLab dataset and compared with the models in [24,25,45], as shown in Table 5. Using the augmented MKLab dataset for model training, the mIoU of the U-Net, LinkNet, PSPNet, DeepLabv2, and Deeplabv3 models increased by 2.43%, 1.91%, 4.53%, 7.1%, and 2.44%, respectively. The data augmentation methods, including flipping, shifting, and rotating from [24,25,45] were utilized. However, ToZero FMNet, which combines U-Net with FMNet and the threshold segmentation algorithm, did not achieve a better detection performance, with an mIoU of only 61.90%. By using the GP loss function, CoAtNet-0 and EfficientNetv2 achieved mIoUs greater than 70% and the detection performance of ships and look-alikes significantly improved compared to the U-Net model. The Ensemble model trained by U-Net and DeepLabv3+ reached an mIoU of 71.12%. The proposed FA-MobileUNet model achieved the best detection performance, with an mIoU of 80.55% and 14.9 M parameters. The proposed model used the pre-trained backbone network of MobileNetv3, which has also been proven to reduce the computational burden while still effectively extracting semantic features for model training.

Among the five marine categories, sea surface and land were effectively detected, as shown in Table 5. Therefore, the detection performance of oil spills and look-alikes was further examined in the experiment to compare the effectiveness of the networks presented in Table 5. The results are summarized in Table 6. In the experiments, the threshold values were set to between 0.5 and 0.7; if the IoU exceeded this value, the target detection results were considered correct. When the threshold was set to 0.7, the proposed model achieved an F1-score of 0.7692 in oil spill detection, which was 0.1127 higher than the DeepLabv3+ model with the second highest F1-score in Table 6. The proposed model achieved an F1-score of over 0.9 in oil spill and look-alike detection when the threshold was set to 0.6. The FA-MobileUNet model consistently achieved the highest F1-score under the different threshold values. Compared with other networks, the proposed model could provide a higher computing efficiency and better oil spill detection performance. The experimental results verified the superior performance of the proposed model.

### 3.5. Oil Spill Detection Result Verification

**Figure 10:**
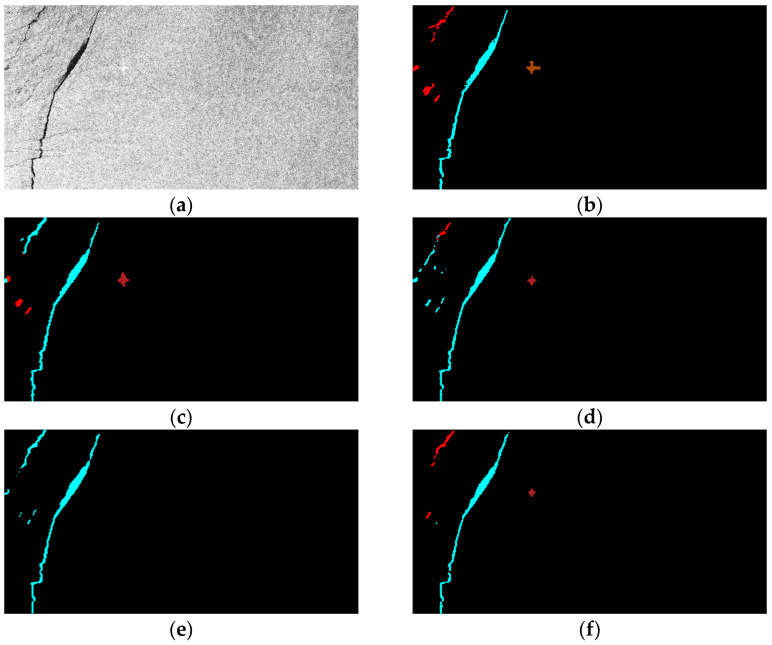

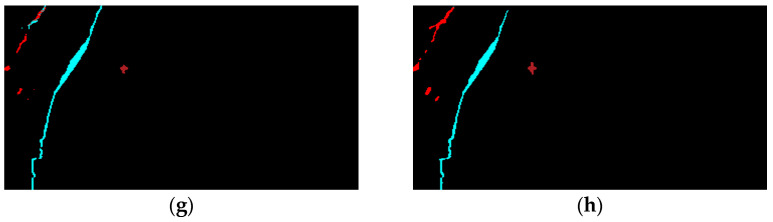
The segmentation results of the 55th image in the MKLab dataset: (**a**) original SAR image, (**b**) the corresponding ground truth data, and results from (**c**) U-Net model, (**d**) LinkNet model, (**e**) PSPNet model, (**f**) DeepLabv2 model, (**g**) DeepLabv3+ model, (**h**) FA-MobileUNet model. Black, cyan, red, brown, and green represent the sea surface, oil spills, look-alikes, ships, and land, respectively.

**Figure 11:**
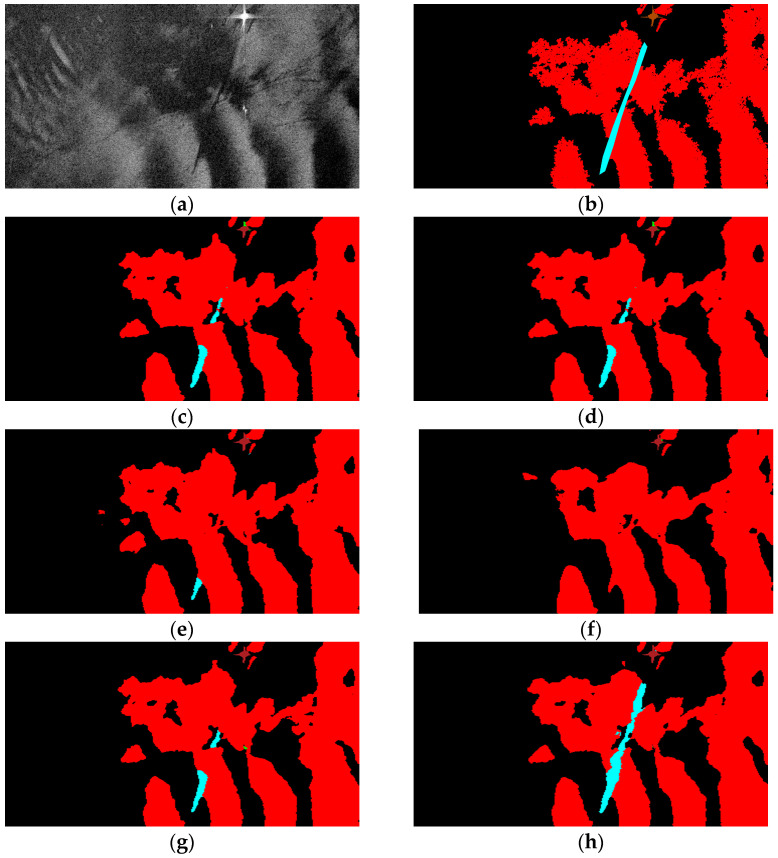
The segmentation results of 71st image in the MKLab dataset: (**a**) original SAR image, (**b**) the corresponding ground truth data, and results from (**c**) U-Net model, (**d**) LinkNet model, (**e**) PSPNet model, (**f**) DeepLabv2 model, (**g**) DeepLabv3+ model, (**h**) FA-MobileUNet model. Black, cyan, red, brown, and green represent the sea surface, oil spills, look-alikes, ships, and land, respectively.

**Figure 12:**
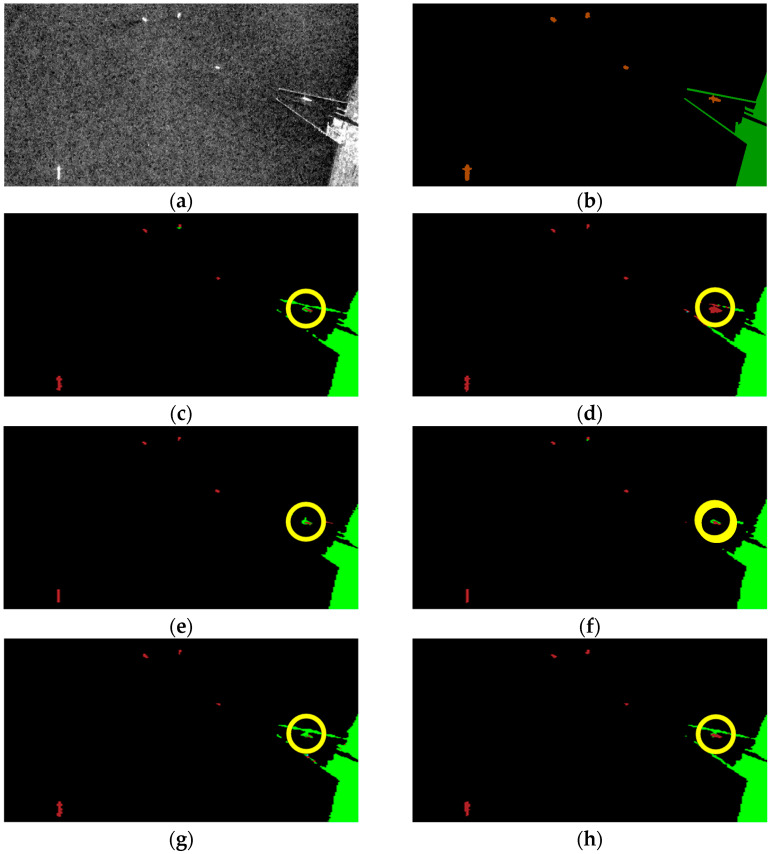
The segmentation results of 106th image in the MKLab dataset: (**a**) original SAR image, (**b**) the corresponding ground truth data, and results from (**c**) U-Net model, (**d**) LinkNet model, (**e**) PSPNet model, (**f**) DeepLabv2 model, (**g**) DeepLabv3+ model, (**h**) FA-MobileUNet model. Black, red, brown, and green represent the sea surface, look-alikes, ships, and land, respectively.

Finally, the segmentation results of some visual samples are depicted in Figure 10, Figure 11 and Figure 12, which were used to qualitatively evaluate the oil spill detection performance of the proposed model. The oil spill images sampled from the testing data were detected by the original U-Net, LinkNet, PSPNet, DeepLabv2, DeepLabv3+, and proposed FA-MobileUNet models. The black, cyan, red, brown, and green colors represent the sea surface, oil spills, look-alikes, ships, and land, respectively. As shown in Figure 10, all models correctly detected oil spills. The U-Net, LinkNet, and PSPNet models misclassified the look-alikes as oil spills. The DeepLabv2 model only detected look-alikes in two regions, while the DeepLabv3+ model had incomplete detection of one look-alike region. However, the FA-MobileUNet model accurately detected all look-alike regions. According to the ground truth data presented in Figure 11b, the oil spills overlapped with the look-alikes. The DeepLabv2 model failed to detect the oil spill regions. Moreover, only the FA-MobileUNet model detected all the oil spill regions, while other models failed to detect some oil spills in overlapping areas. As shown in Figure 12a, there were five ships in the SAR image, one of which was located inside the port. All models effectively detected ships outside the port area. However, for the ship inside the port, the U-Net and DeepLabv3+ models incorrectly classified the ship into the land category, because the ship was close to the breakwater, as shown in the yellow circled area in Figure 12c,g. While the LinkNet model correctly identified the ship, it incorrectly identified part of the breakwater as the ship category. Moreover, the PSPNet and DeepLabv2 models not only misclassified the ship but also failed to detect the breakwater. As shown in Figure 12h, the FA-MobileUNet model correctly detected all ships in the image. The experimental results showed that the FA-MobileUNet model had an improved multi-scale and multi-target detection performance, resulting in more accurate segmentation results compared to the other segmentation networks.

## 4. Discussion

### 4.1. Oil Spill Detection Performance Analysis

**Figure 13:**
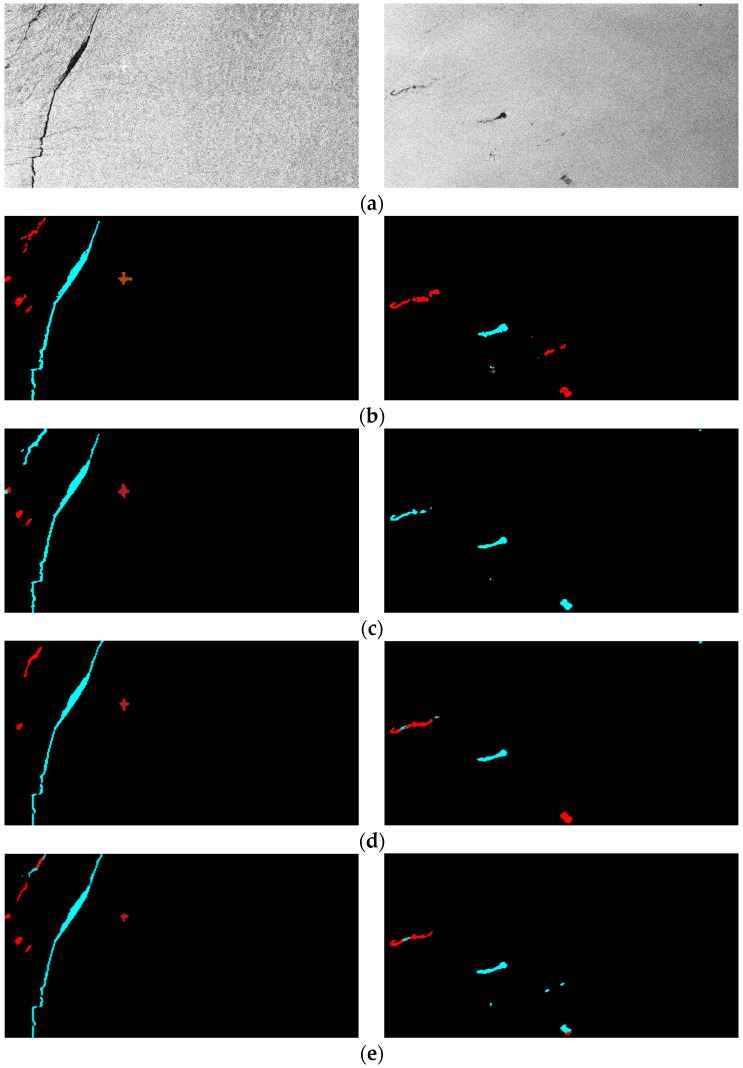

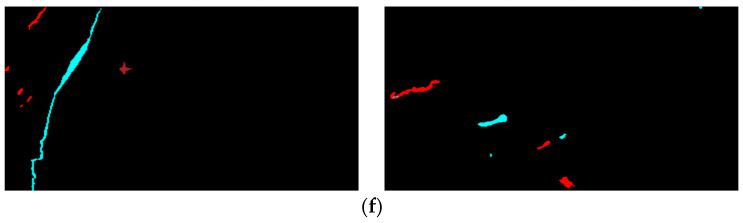
The segmentation results of U-Net model with different modules: (**a**) original SAR image, (**b**) the corresponding ground truth data, and results from (**c**) U-Net model, (**d**) U-Net model with CBAM, (**e**) U-Net model with ASPP module, (**f**) U-Net with FA module. Black, cyan, red and brown represent the sea surface, oil spills, look-alikes and ships, respectively.

Oil spills and look-alikes are somewhat competitive because dark areas identified as oil spills could be misclassified as look-alikes and vice versa. This phenomenon complicates the distinction between oil spills and look-alikes, as shown in Section 3.4. The detection models [31] provided a relatively high performance for the oil spills, but had a poor detection performance for look-alikes. While the models [25,45] improved the overall detection performance, the oil spill detection performance was not improved compared to the original U-Net model. Therefore, this study analyzed the improvement in oil spill detection performance of the three modules, including CBAM, ASPP, and FA, as shown in Table 7. These modules increased the IoU of oil spills by more than 7%. By effectively aggregating high-level semantic features and low-level spatial information, the detection performance of look-alikes with diverse scales in the images can be improved. Therefore, the U-Net model with the FA module achieved a better look-alike detection performance, reaching an IoU of over 70%. These modules helped to correctly classify dark areas as oil spills or look-alikes. The segmentation results of the U-Net model with different module combinations are shown in Figure 13. The detection results of the original U-Net model misclassified the dark areas, as shown in Figure 13c. The U-Net model with the CBAM enhanced the spatial information and helped reduce the misclassification of dark areas. The U-Net model combined with the ASPP module could detect targets with different scales. Finally, the U-Net model with the FA module effectively aggregated multi-scale contextual information, thereby achieving a better detection performance. Therefore, the U-Net model combined with different modules can improve the feature extraction of spatial information and semantic features of the network, which reduced the fragmentation and misclassification of dark areas.

### 4.2. Revised Labeled Data for Ships

The source of the oil spill dataset used in the study was the Sentinel-1 data, with a swath that is 250 km wide and a pixel spacing of 10 m × 10 m. SAR sensors with such specifications can cover a wide area of interest while capturing relatively small-sized ship targets. As shown in Table 5, the deep learning methods of [24,25,31,45] had poor detection performance for ships. The experimental results showed that the proposed model can effectively extract features of ships, with an mIoU of over 60%. Moreover, the proposed FA-MobileUNet model demonstrated effective detection of ships, successfully distinguishing ships close to land, as shown in Figure 12. This study made full use of features extracted from the encoding layer at different scales and aggregated them into the decoding layer to effectively improve the overall detection performance of the oil spill dataset.

**Figure 14:**
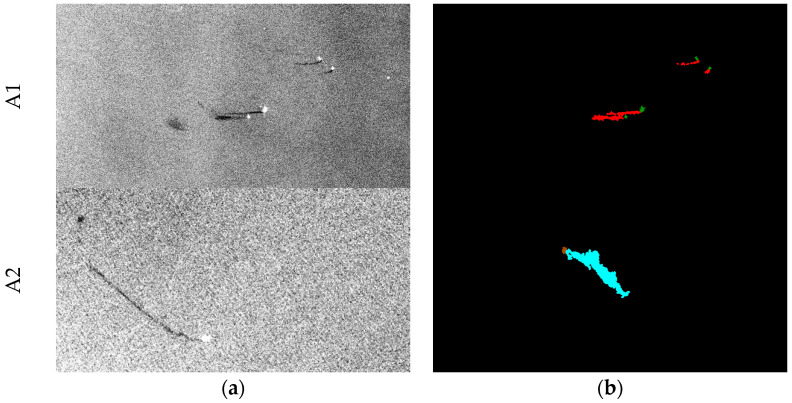
The incorrect ground truth data in the dataset. A1 and A2 are the 111th and 275th training images, respectively. Black, cyan, red, brown, and green represent the sea surface, oil spills, look-alikes, ships, and land, respectively. (**a**) SAR image. (**b**) Ground truth data.

**Figure 15:**
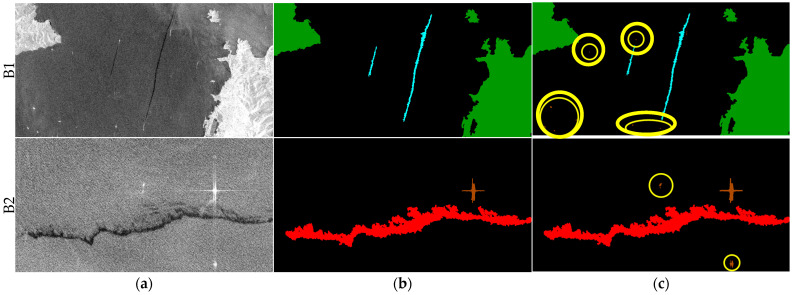
Example of the revised ground truth data in the dataset. B1 and B2 are the 140th and 157th training images, respectively. Black, cyan, red, brown, and green represent the sea surface, oil spills, look-alikes, ships, and land, respectively. (**a**) SAR image. (**b**) Original ground truth data. (**c**) Revised ground truth data.

Furthermore, the experimental results in Table 4 and Table 5 show that the detection performance for ships was the worst among the five categories. Therefore, this study identified the reason by checking the ground truth data of the MKLab dataset. Although the data were collected based on oil spill events, some errors in the manual annotations were found. In the MKLab dataset, some annotated categories in the images are wrong or some ground truth data corresponding to SAR images are incorrect. For instance, the brown color representing ships was incorrectly annotated as the green color representing land, as shown in A1 of Figure 14. The image and its corresponding labeling data are completely incorrect, as shown in A2 of Figure 14. Moreover, the poor ship detection performance may be due to the fact that many ships in the training images were not annotated, resulting in the inability to effectively detect the ships in the testing data. Therefore, this study re-examined the augmented MKLab dataset and corrected the ground truth data for a total of 83 images with incorrect or missing annotations; some of these samples are shown in Figure 15.

The experiment compared the detection performance of the deep learning models using the augmented and revised MKLab datasets, as shown in Table 8. The U-Net, LinkNet, PSPNet, DeepLabv2, DeepLabv3+, and FA-MobileUNet models were utilized to evaluate the revised MKLab dataset. Compared with the results using the augmented MKLab dataset, the ship detection performance of the models trained by the revised MKLab dataset improved, and the IoU increased by 8.77%, 12.15%, 25.86%, 26.15%, 15.94%, and 14.72% for the U-Net, LinkNet, PSPNet, DeepLabv2, DeepLabv3+, and FA-MobileUNet models, respectively. With the exception of the ship category, only a slight difference in the detection performance was observed because the revised MKLab dataset primarily labeled the missing annotation of ships in the dataset. By correcting these ground truth data, the ship category can be more effectively learned by the deep learning network. The experimental results validated that the proposed method outperforms the semantic segmentation models proposed in other studies in terms of detection performance.

## 5. Conclusions

This study proposed an oil spill detection method based on the U-Net model using SAR data. The proposed FA-MobileUNet model utilizes the lightweight pre-trained MobileNetv3 network as the backbone. Additionally, the CBAM and ASPP modules are employed to efficiently extract the semantic features. Finally, the multi-scale feature maps from the encoding layer are aggregated to the decoding layer, allowing the deep learning model to effectively learn the features of each category. The performance of the proposed method was evaluated through experiments on the MKLab dataset. In addition, SAR images of oil spill events from 2015 to 2022 were collected to augment the training data of the MKLab dataset. The oil spill detection performance was assessed using IoU. The experimental results demonstrated that the proposed FA-MobileUNet model outperforms other models in terms of computation efficiency and detection performance. The proposed model achieved an mIoU of over 80%, with only 48% of the parameters of the original U-Net model. The experiments also validated that the proposed model can better distinguish between look-alikes and oil spills. Moreover, the detection performance for ships was greatly improved by aggregating multi-scale features. The study also identified the reasons for the poor detection performance of ships in other deep learning algorithms using the MKLab dataset. Adjusting incorrect and missing annotations in the dataset allowed the deep learning model to be trained properly for each category. In conclusion, the proposed model achieved a high detection performance and computational efficiency on the oil spill dataset.

## Figures and Tables

**Table 1:** Five categories and their corresponding labels.

Category	1D Label	RGB Label
Sea surface	0	Black
Oil spills	1	Cyan
Look-alikes	2	Red
Ships	3	Brown
Land	4	Green

**Table 2:** Detection performance of U-Net model with all possible combinations of CBAM, ASPP, and FA.

Baseline	CBAM	ASPP	FA	Parameters (M)	mIoU (%)
✓				31.03	72.19
✓	✓			31.12	75.07
✓		✓		39.49	76.58
✓			✓	31.40	79.14
✓	✓	✓		39.58	78.42
✓	✓		✓	31.49	80.15
✓		✓	✓	39.86	80.81
✓	✓	✓	✓	40.42	82.37

**Table 3:** Detection performance of U-Net model with different backbone architectures.

Model	Backbone	Parameters (M)	mIoU (%)
U-Net	x	31.03	72.19
	VGG16	25.86	72.73
	VGG19	28.81	72.82
	ResNet50	20.67	67.21
	DenseNet121	16.41	74.77
	EfficientNetB0	10.83	66.57
	Inceptionv3	17.66	75.22
	MobileNetv2	11.75	73.60
	MobileNetv3	10.57	75.98

**Table 4:** Detection performance evaluation of the proposed method using the original MKLab dataset in terms of IoU (%).

Model	Backbone	Parameters	Sea Surface	Oil Spills	Look-Alikes	Ships	Land	mIoU
FA-MobileUNet	MobileNetv3	14.9 M	96.98	70.42	70.15	60.21	96.91	78.93
U-Net	ResNet-101	51.5 M	93.90	53.79	39.55	44.93	92.68	64.97
LinkNet	ResNet-101	47.7 M	94.99	51.53	43.24	40.23	93.97	64.79
PSPNet	ResNet-101	3.8 M	92.78	40.10	33.79	24.42	86.90	55.60
DeepLabv2	ResNet-101	42.8 M	94.09	25.57	40.30	11.41	74.99	49.27
DeepLabv3+	MobileNetv2	2.1 M	96.43	53.38	55.40	27.63	92.44	65.06

**Table 5:** Detection performance comparison of the proposed method using the augmented MKLab dataset with other segmentation networks in terms of IoU (%).

Model	Backbone	Parameters	Sea Surface	Oil Spills	Look-Alikes	Ships	Land	mIoU
FA-MobileUNet	MobileNetv3	14.9 M	97.62	74.28	72.96	61.47	96.44	80.55
U-Net	ResNet-101	51.5 M	95.47	57.01	44.82	46.62	93.08	67.40
LinkNet	ResNet-101	47.7 M	94.82	52.95	47.52	45.11	93.12	66.70
PSPNet	ResNet-101	3.8 M	93.03	45.65	40.62	30.25	91.12	60.13
DeepLabv2	ResNet-101	42.8 M	95.02	43.12	46.23	15.12	82.34	56.37
DeepLabv3+	MobileNetv2	2.1 M	96.57	56.34	57.06	32.92	94.18	67.41
ToZero FMNet	x	36.0 M	94.53	49.95	41.40	25.44	87.11	61.90
CoAtNet-0	x	29.4 M	95.40	50.22	58.85	69.09	94.49	73.61
EfficientNetv2	B1	16.7 M	95.19	56.42	62.23	72.80	96.59	76.65
Ensemble Model	x	x	96.78	56.10	58.88	47.28	96.59	71.12

**Table 6:** Detection performance comparison of the proposed method using the augmented MKLab dataset with other segmentation networks in terms of F1-score.

	IoU > 0.7		IoU > 0.6		IoU > 0.5	
	Oil Spills	Look-Alikes	Oil Spills	Look-Alikes	Oil Spills	Look-Alikes
FA-MobileUNet	0.7692	0.8542	0.9268	0.9524	0.9708	0.9815
U-Net	0.5574	0.5750	0.7429	0.7191	0.8790	0.8485
LinkNet	0.4833	0.6000	0.6715	0.7826	0.8105	0.9333
PSPNet	0.3019	0.4878	0.5124	0.6222	0.6667	0.7216
DeepLabv2	0.2593	0.5412	0.3652	0.6809	0.5426	0.7347
DeepLabv3+	0.6565	0.6250	0.8571	0.8172	0.9398	0.9524

**Table 7:** Performance comparison of the different modules in terms of IoU (%).

Model	Modules	Oil Spills	Look-Alikes	Ships
U-Net	×	59.14	54.78	55.31
	+CBAM	66.85	58.42	57.64
	+ASPP	68.48	62.24	57.61
	+FA	69.92	73.02	59.68

**Table 8:** Performance comparison of the augmented and revised MKLab datasets in terms of IoU (%).

Model	MKLab Dataset	Sea Surface	Oil Spills	Look-Alikes	Ships	Land	mIoU
FA-MobileUNet	Augmented	97.62	74.28	72.96	61.47	96.44	80.55
	Revised	97.54	75.85	72.67	76.19	96.48	83.74
U-Net	Augmented	95.47	57.01	44.82	46.62	93.08	67.40
	Revised	95.54	56.91	47.12	55.39	94.24	69.84
LinkNet	Augmented	94.82	52.95	47.52	45.11	93.12	66.70
	Revised	94.77	53.06	46.87	57.26	93.81	69.15
PSPNet	Augmented	93.03	45.65	40.62	30.25	91.12	60.13
	Revised	93.25	45.67	40.24	56.11	92.03	65.46
DeepLabv2	Augmented	95.02	43.12	46.23	15.12	82.34	56.37
	Revised	94.28	44.31	45.94	41.27	82.65	61.69
DeepLabv3+	Augmented	96.57	56.34	57.06	32.92	94.18	67.41
	Revised	96.28	56.22	56.12	48.86	94.82	70.46

## Data Availability

The data presented in this study are available upon request from the authors. The data are not publicly available due to privacy restrictions.
